# The ganglioside antigen G_D2_ is surface-expressed in Ewing sarcoma and allows for MHC-independent immune targeting

**DOI:** 10.1038/bjc.2012.57

**Published:** 2012-02-28

**Authors:** S Kailayangiri, B Altvater, J Meltzer, S Pscherer, A Luecke, C Dierkes, U Titze, K Leuchte, S Landmeier, M Hotfilder, U Dirksen, J Hardes, G Gosheger, H Juergens, C Rossig

**Affiliations:** 1 Department of Pediatric Haematology and Oncology, University Children's Hospital Muenster, Albert-Schweitzer-Campus 1, Muenster, 48149, Germany; 2 Gerhard-Domagk Institute of Pathology, University of Muenster, Albert-Schweitzer-Campus 1, Muenster, 48149, Germany; 3 Department of Orthopedic Surgery, University Hospital Muenster, Albert-Schweitzer-Campus 1, Muenster, 48149, Germany

**Keywords:** Ewing sarcoma, G_D2_, cellular immunotherapy, gene transfer, cancer targets

## Abstract

**Background:**

Novel treatment strategies are needed to cure disseminated Ewing sarcoma. Primitive neuroectodermal features and a mesenchymal stem cell origin are both compatible with aberrant expression of the ganglioside antigen G_D2_ and led us to explore G_D2_ immune targeting in this cancer.

**Methods:**

We investigated G_D2_ expression in Ewing sarcoma by immunofluorescence staining. We then assessed the antitumour activity of T cells expressing a chimeric antigen receptor specific for G_D2_ against Ewing sarcoma *in vitro* and *in vivo*.

**Results:**

Surface G_D2_ was detected in 10 out of 10 Ewing sarcoma cell lines and 3 out of 3 primary cell cultures. Moreover, diagnostic biopsies from 12 of 14 patients had uniform G_D2_ expression. T cells specifically modified to express the G_D2_-specific chimeric receptor 14. G2a-28*ζ* efficiently interacted with Ewing sarcoma cells, resulting in antigen-specific secretion of cytokines. Moreover, chimeric receptor gene-modified T cells from healthy donors and from a patient exerted potent, G_D2_-specific cytolytic responses to allogeneic and autologous Ewing sarcoma, including tumour cells grown as multicellular, anchorage-independent spheres. G_D2_-specific T cells further had activity against Ewing sarcoma xenografts.

**Conclusion:**

G_D2_ surface expression is a characteristic of Ewing sarcomas and provides a suitable target antigen for immunotherapeutic strategies to eradicate micrometastatic cells and prevent relapse in high-risk disease.

The outcome in patients with disseminated Ewing sarcoma remains poor despite intensive multimodal treatment regimens (Ladenstein *et al*, 2010). The disease often responds well to chemotherapy, but systemic relapses occur in the majority of patients. Targeting of residual disease by novel treatment strategies may sustain remission and improve outcome. In some cancers, tumour antigen-specific antibodies (Coiffier *et al*, 2010; Yu *et al*, 2010) or T cells (Dudley *et al*, 2002) have shown promising activity. Immune targeting of Ewing sarcoma has been limited by the lack of adequate antigens. Although the breakpoint region of the characteristic EWS-FLI1 fusion protein contains unique peptide sequences specifically recognised by T cells (Meyer-Wentrup *et al*, 2005), clinically tested fusion peptides failed to induce high-avidity T-cell responses (Mackall *et al*, 2008).

Recent experimental evidence supports either primitive neural crest cells (Coles *et al*, 2008) or mesenchymal stem cells (MSCs) (Castillero-Trejo *et al*, 2005; Riggi *et al*, 2005; Tirode *et al*, 2007) as candidate cells of origin for Ewing sarcoma. Both hypotheses can be reconciled by the recent finding that MSCs can arise from neural crest progenitors (Takashima *et al*, 2007). A shared feature of both neural crest and MSCs is expression of the ganglioside antigen G**_D2_** (Martinez *et al*, 2007; Xu *et al*, 2009). Individual reports describe variable levels of G_D2_ expression on Ewing sarcoma cell lines (Lipinski *et al*, 1987) and in rare individual primary Ewing sarcoma samples (Cheung *et al*, 1987; Heiner *et al*, 1987). A systematic study of G_D2_ expression in Ewing sarcoma has not yet been performed.

G_D2_ is a cell-surface molecule with a highly restricted pattern of expression and therefore amenable to targeting by monoclonal antibodies (mAbs) and their derivatives. In high-risk neuroblastoma, a highly G_D2_-expressing malignancy, immunotherapy with a chimeric G_D2_-specific antibody combined with GM-CSF and IL-2 significantly improved event-free survival (Yu *et al*, 2010). An alternative strategy is based on the recruitment of T-cell immune responses to G_D2_ by expression of recombinant chimeric antigen receptors (CARs). Chimeric antigen receptors consist of the antigen-binding domain of a mAb linked to T-cell receptor signalling domains. Chimeric antigen receptor-engineered G_D2_-specific T cells efficiently interact with G_D2_-expressing neuroblastoma cells *in vitro*, resulting in specific tumour cytolysis (Rossig *et al*, 2001). In a clinical trial in patients with refractory and relapsed disease, G_D2_-specific T-cell transfer was well tolerated, and first evidence of *in vivo* persistence and antitumour activity was obtained (Pule *et al*, 2008; Louis *et al*, 2011). Here, we hypothesised that G_D2_ expression may be a common characteristic of Ewing sarcomas and may provide a therapeutic target in this disease.

## Material and methods

### Cell lines

The identity of all cancer cell lines was confirmed by short tandem repeat profiling (Supplementary Figure S1). The Ewing sarcoma cell lines RD-ES, SK-ES-1, TC-71 and Cado-ES-1 were from DSMZ (Braunschweig, Germany). VH-64 cells after multiple and after only 3 *in vitro* passages (VH-64.P3) following isolation from a malignant pleural effusion, and WE-68 cells were gifts from Frans van Valen's laboratory at the Institute of Experimental Orthopedics of University of Muenster, Germany. These cell lines were characterised by the EuroBoNeT consortium (Ottaviano *et al*, 2010). A-4573, TC-32, TTC-466, and 5838 were from the cell line bank at Children's Hospital Los Angeles. LAN-1 and LAN-5 (provided by Robert Seeger, Los Angeles, CA, USA), and JF (Malcolm K Brenner, Houston, TX, USA) are human neuroblastoma cell lines. A-204 (DSMZ) is a rhabdomyosarcoma cell line. The packaging cell lines Phoenix-ampho and FLYRD18 were provided by Gary P Nolan (Stanford, CA, USA) and E Vanin (Houston, TX, USA), respectively. Normal human fibroblasts generated from skin biopsies were obtained from Cliona Rooney (Houston, TX, USA). For standard adherent growth, tumour cells were cultured in collagen-coated 25 cm^2^ tissue culture flasks (VH-64, WE-68, Cado-ES-1, RD-ES, SK-ES-1, TTC-466) or in uncoated flasks (all others) in RPMI 1640 medium (Invitrogen, Darmstadt, Germany), supplemented with 10% heat-inactivated fetal calf serum (FCS; Thermo Fisher, Bonn, Germany) and 2 mM L-glutamine and maintained at 37 °C and 5% CO_2_. To generate primary Ewing sarcoma cell cultures, biopsy material from metastatic relapse tumours in two adolescent patients (MS-PES-1, MS-PES-3) was dissected into 1–2 mm fragments, incubated in trypsin (0.05%)/EDTA (0.02%) solution (PAA, Cölbe, Germany), and passed through a cell strainer. Single-cell suspensions were cultured on collagen-coated plates, and adherent cells were re-established in secondary culture.

### Patients and tumour samples

Tumour samples were obtained from diagnostic biopsies performed in the Department of Orthopedic Surgery in Muenster in a consecutive cohort of patients treated according to the EURO E.W.I.N.G-99 study protocol. The single selection criterion was availability of frozen samples. Diagnosis was confirmed by immunohistochemistry staining of CD99 and molecular detection of a characteristic fusion transcript (Table 1). Approval for using tumour and/or peripheral blood samples from patients and healthy donors was obtained from the University of Muenster Ethical Board, and informed consent was obtained in accordance with the Declaration of Helsinki.

### Constructs

Generation of the G_D2_-specific CAR 14.G2a-28*ζ* is described in detail in previous publications from our group (Rossig *et al*, 2001; Altvater *et al*, 2006). The receptor contains the single-chain antibody domain of the monoclonal anti-G_D2_ antibody 14.G2a (Mujoo *et al*, 1989), the transmembrane domain of CD8*α*, and the intracellular domains of CD28 and TCR*ζ*. Antigen specificity of 14.G2a CAR gene-modified T cells for G_D2_ was confirmed in antibody and cold target inhibition assays (Rossig *et al*, 2001, 2002). A published construct with FMC-63 mAb-derived CD19-specificity was used for comparative experiments (Rossig *et al*, 2006). Both *CAR* genes were subcloned into the *Bam*HI and *Nco*I sites of the retroviral vector SFG (provided by RC Mulligan, Cambridge, MA, USA).

### Production of recombinant retrovirus, transduction and expansion of T cells

Fresh retroviral supernatants collected from transiently transfected Phoenix ampho cells were used to infect the packaging cell line FLYRD18, and viral supernatants were generated by adding Isocoves-modified Dulbecco medium (Invitrogen) supplemented with 20% FCS for 24 h of incubation at 32 °C. T cells were nonspecifically prestimulated with CD3- and CD28-specific antibodies and retrovirally transduced with the CAR genes as previously described (Rossig *et al*, 2001; Altvater *et al*, 2006).

### Flow cytometry

Ewing sarcoma cells were stained with fluorescence-conjugated mAbs directed against G_D2_ (14G2a; BD Pharmingen, Heidelberg, Germany), CD166 (mAb 1172, R&D, Wiesbaden, Germany), and CD99 (BD Pharmingen). After fluorescein isothiocyanate (FITC)-labelled 14.G2a mAb became commercially unavailable, further analyses were performed using unlabelled 14.G2a, followed by FITC labelling using secondary goat anti-mouse Ab (both from BD Pharmingen). T cells were analysed using mAbs against CD3, CD8, CD4, and CD56 (BD Pharmingen). Surface expression of 14.G2a-28*ζ* was determined by staining with a biotinylated goat anti-mouse mAb specific for IgG F(ab’)2 fragment (Jackson ImmunoResearch, Cambridgeshire, UK) and secondary phycoerythrin-labelled streptavidin antibody (BD Pharmingen). For each sample, 20 000 cells were analysed with FACS Calibur and BD Cell Quest Software or with FACS Canto and FACS Diva Software. Relative fluorescence intensities (RFI) were calculated by dividing mean fluorescence intensities of mAb-stained cells by those obtained with isotype antibodies or in the absence of antibody.

### Immunohistochemistry

Cryostat-frozen tumour sections of 4 *μ*m were fixed in 2% paraformaldehyde for 10 min, then permeabilised for 3 h in phosphate-buffered saline (PBS) containing 0.075% Tween and 1% bovine serum albumin. Sections were then incubated overnight with FITC-conjugated 14.G2a mAb, diluted 1 : 50 (2 *μ*g ml^–1^) in PBS supplemented with 0.01% Tween, and counterstained with DAPI. As controls, cryostat sections of primary neuroblastoma tumours and of pelleted G_D2_^+^ and G_D2_-negative tumour cell lines were stained by immunohistochemistry as above. Stained sections were independently reviewed by two pathologists and compared with a hematoxylin–eosin-stained slide from each case.

### Analysis of cytokine production and granzyme B secretion

To assess target-induced cytokine production, T cells were seeded at 1 × 10^6^ cells per well in a 24-well plate and stimulated with 1 × 10^6^ irradiated tumour target cells for 6 h. Cytokine secretion was blocked with 10 *μ*g brefeldin A (Sigma, Munich, Germany) per 2 × 10^6^ cells for the final 4 h of co-incubation. Cells were permeabilised using a proprietary solution (Becton Dickinson, San Jose, CA, USA), and then stained with interferon (IFN)-*γ* and tumour necrosis factor (TNF)-*α*-specific antibodies according to the manufacturer's recommendations. For IFN-*γ* and granzyme B ELISpot analysis, 10 000 T cells per well were plated in triplicate and stimulated overnight with 50 000 tumour cells per well on Multiscreen 96-well plates (Millipore, Schwalbach, Germany) coated with 10 *μ*g ml^–1^ anti-IFN-*γ* Ab or 15 μg ml^–1^ anti-granzyme B mAb, then incubated with the respective capture antibodies and analysed following the instructions of the human IFN-*γ* and granzyme B ELISpot kits (both by Mabtech AB, Hamburg, Germany). Spots were counted using an automated reader (CTL ImmunoSpot S5 UV Analyser, CTL Europe, Bonn, Germany).

### Cytotoxicity assays

For ^51^Cr release assays, T effector cells were co-incubated in triplicate with 2500 target cells labelled with 100 *μ*Ci^51^Cr/1 × 10^6^ cells (PE Applied Biosystems; Weiterstadt, Germany) in a total volume of 200 *μ*l in a V-bottomed 96-well plate at 37 °C and 5% CO_2_. Supernatants were harvested and radioactivity was counted in a gamma counter. Maximum release was determined by target cell lysis with Triton X. For 16-h cytotoxicity experiments, target cells were co-incubated at 1 × 10^5^ cells per well in 96-well plates with various numbers of T cells for 16 h. The percentage of viable tumour cells was determined by co-staining with CD99-, CD3-, and CD45-specific mAbs and subsequent flow cytometry analysis of the relative proportions of cells within the CD99+CD3−CD45− tumour cell gate.

### Culture and analysis of spheres

Tumour cells from monolayer cultures were seeded at 2000 cells per well in Ultra-low attachment six-well plates (Costar, Corning, NY, USA). The serum-free sphere culture medium consisted of DMEM/F12 (1 : 1) supplemented with 2% B27 (reagents from Invitrogen), 20 ng ml^–1^ recombinant human epidermal growth factor (Strathmann Biotech, Hamburg, Germany), 20 ng ml^–1^ leukaemia inhibitory factor (20 ng ml^–1^), and 10 IE ml^–1^ (5 *μ*g ml^–1^) heparin (Roche, Mannheim, Germany). Recombinant human epidermal growth factor was added once on day 4. To assess the sensitivity of spheres to G_D2_-retargeted T-cell lysis, intact spheres on day 9 were transferred into individual wells of 96-well round bottom plates in sphere culture medium. Maximum perpendicular sphere diameters were quantified using a Zeiss Observer Z1 inverted microscope and Axiovision 4.8 imaging software (Carl Zeiss, Goettingen, Germany) and used to calculate sphere volumes. For each experiment, 27 similar-sized spheres with a volume of ∼200 *μ*m^3^ were selected and co-incubated with 1000 14.G2a-28*ζ* T cells or non-transduced T cells per sphere, or in the presence of medium alone for 16 h. Triplicates of three pooled spheres each were used for analysis. Spheres were manually dissociated in an enzyme-free solution containing 1 mM EDTA, 40 mM Tris-HCl and 150 mM NaCl, and viable cells within the tumour cell gate were quantified as above.

### Xenogeneic NOD/scid mouse model of Ewing sarcoma

Mouse experiments were approved by the animal care committee of the local government (Bezirksregierung Muenster, Muenster, Germany, Az. 87-51.04.2010.A117). Eight to twelve-week old NOD/scid mice (Charles River Laboratories, Sulzfeld, Germany) were irradiated with a single dose of 3.5 Gy from a linear accelerator 1 day before transplantation to remove residual NK cell activity (Vormoor *et al*, 2001). A total of 5 × 10^6^ tumour cells were injected subcutaneously into the right flank. Palpable tumours were treated by five intratumoural injections of 1 × 10^7^ 14.G2a-28*ζ*-transduced T cells, or non-transduced T cells as controls each over 2 weeks. Tumour growth was monitored by caliper quantification of diameters.

### Statistical analysis

The student *t*-test was used to test for significance in each set of values, assuming equal variance. Mean values±s.d. are given unless otherwise stated.

## Results

### G_D2_ is expressed on the surface of Ewing sarcoma cell lines and primary Ewing sarcoma cells

**Figure 1:**
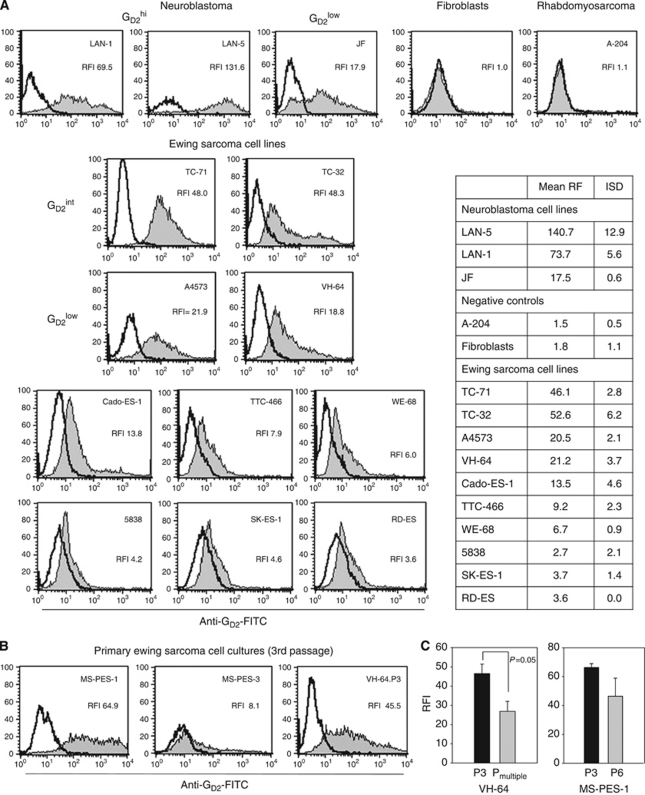
Expression of G_D2_ on various cell lines and primary tumour cell cultures by flow cytometry. (**A**) G_D2_ expression was analysed in various cell lines by flow cytometry after staining with FITC-labelled 14.G2a antibody. According to the surface expression level, cell lines were categorised as highly (G_D2_^high^), intermediately (G_D2_^int^), and low (G_D2_^low^) G_D2_-expressing cell lines. Controls were unstained and/or isotype-stained cells. The table shows the mean RFI and s.d. of two to four individual experiments. (**B**) Primary Ewing sarcoma cell cultures established from tumour biopsies upon disseminated Ewing sarcoma relapse in two patients and an early passage of VH-64 were analysed by flow cytometry as above. (**C**) Surface G_D2_ expression was directly compared in VH-64 cells after three (P3) and after multiple (P_multiple_) *in vitro* culture passages, and in MS-PES-1 cells after three (P3) *vs* six (P6) passages. Standard error bars are derived from three independent experiments performed on individual days.

Surface G_D2_ expression was detected by flow cytometry using mAb 14. G2a in all Ewing sarcoma cell lines (Figure 1A). In comparison with the RFI of 17.5±0.6 (JF) to 140.7±12.9 (LAN-5) in neuroblastoma cells, expression densities in Ewing sarcoma cells ranged between RFIs of 2.7±2.1 and 52.6±6.2. As expected, primary human fibroblasts and rhabdomyosarcoma cell lines were G_D2_-negative.

To exclude that the 14.G2a reactivity of Ewing sarcoma cell lines was due to CD166 rather than G_D2_ expression, as suggested in one report (Wierzbicki *et al*, 2008), we performed additional staining experiments with the CD166-specific mAb 1172. CD166^high^ fibroblasts were not reactive with 14.G2a mAb (Supplementary Figure S2). Thus, compatible with a recent report (Agrawal and Frankel, 2010), 14.G2a does not cross-react with surface epitopes of CD166.

To confirm G_D2_ surface expression in primary Ewing sarcoma cells, we analysed VH-64 cells after only three passages, as well as two cell cultures newly initiated from relapse tumour biopsies (Table 1), (Supplementary Figure S3). G_D2_ was expressed at variable surface densities on all three early Ewing sarcoma cell cultures (Figure 1B). Surface expression of G_D2_ was significantly increased in early *vs* established cultures of VH-64, and the same trend was found in MS-PES-1 cells (Figure 1C).

**Figure 2:**
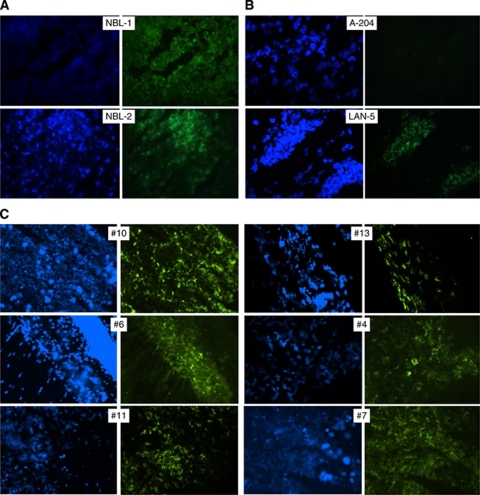
G_D2_ expression in primary Ewing sarcoma tissue sections by immunofluorescence staining with FITC-labelled 14.G2a antibody (blue fluorescence, DAPI, green fluorescence, 14.G2a). Sections through pelleted A-204 rhabdomyosarcoma cells (**A**) were used as negative controls, and LAN-5 neuroblastoma cells (**A**) and primary neuroblastoma tissue sections (**B**) served as positive controls. Details regarding the individual patients are found in Table 1. (**C**) Moderate to intense homogeneous cell membrane staining was found in 12 of 14 Ewing sarcoma tumours, as exemplified by tumour samples from six patients.

Ultimately, we assessed G_D2_ expression in cryopreserved tissue sections obtained at primary diagnosis from 14 additional Ewing sarcoma patients (Table 1). Moderate to intense G_D2_ expression was detected in tumour cells from 12 of the 14 Ewing sarcoma patients by fluorescence microscopy, including patients with localised and metastatic disease (Figure 2C, Table 1). Immunoreactivity had characteristic cell membrane localisation and was restricted to tumour cells, whereas surrounding tissue was negative for G_D2_. Intensity and pattern of staining was comparable to neuroblastoma tissue sections and to LAN-5 cells (Figure 2). Thus, G_D2_ expression is a common characteristic of Ewing sarcoma throughout various disease manifestations including localised, disseminated or relapsed disease.

### G_D2_-specific, CAR-reengineered T cells functionally interact with Ewing sarcoma cells

**Figure 3:**
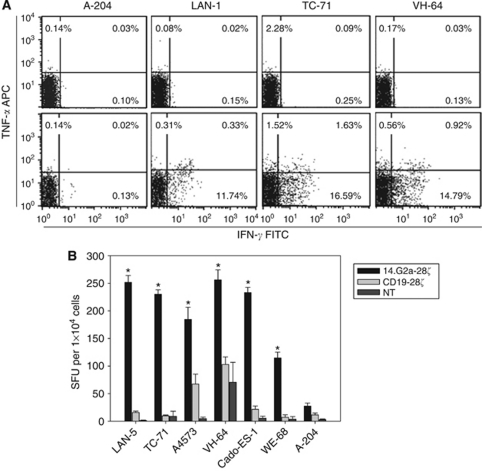
CAR-transduced, G_D2_-specific T cells functionally interact with Ewing sarcoma targets. (**A**) Intracellular production of IFN-*γ* and TNF-*α* by non-transduced (top panel) and 14.G2a-28ζ-transduced (bottom panel) T-cell cultures was quantified by flow cytometry in response to 6-h co-incubation with the G_D2_-negative rhabdomyosarcoma cell line A-204, the G_D2_^high^ neuroblastoma cell line LAN-1, and the Ewing sarcoma cell lines TC-71 (G_D2_^int^) and VH-64 (G_D2_^low^), respectively. Shown is one representative experiment of three. (**B**) IFN-*γ* release by 14.G2a-28ζ-transduced T cells *vs* CD19-28ζ- or non-transduced control T cells in response to exposure to LAN-1 positive control and A-204 negative control cells as well as various Ewing sarcoma cell lines was investigated by ELISpot analysis following overnight co-incubation of effector and target cells. Asterisks indicate significant (*P*<0.05) differences to control. Representative experiment of two.

To investigate whether Ewing sarcoma cells induce antigen-specific activation of G_D2_-specific T cells, T cell cultures from six healthy donors were gene-modified with the G_D2_-specific CAR 14.G2a-28*ζ* (Altvater *et al*, 2006). As Ewing sarcoma cells are highly sensitive to NK cell lysis *in vitro* (Verhoeven *et al*, 2008; Cho *et al*, 2010), NK cells were removed by magnetic cell selection using CD56-specific microbeads. Retroviral transduction of T cells resulted in 14.G2a-28*ζ* surface expression in a median of 70.4±9.8 (range 54.9–84.9) T cells. Gene-modified T cell cultures on day 14 consisted of both CD3+CD8+ (47.8±9.0%) and CD3+CD4+ (44.8±13.0%) T cells, whereas CD3-CD56+ NK cells were <1% in all cultures. Co-incubation of 14.G2a-28*ζ* transduced T cells with the G**_D2_**-positive Ewing sarcoma cell lines TC-71 and VH-64, but not the G_D2_-negative rhabdomyosarcoma cell line A-204 induced production of IFN-*γ* and TNF-*α* (Figure 3A). Cytokine production in response to both Ewing sarcoma cell lines was comparable to the neuroblastoma cell line LAN-1 that expresses G_D2_ at high density and is widely used for preclinical evaluation of G_D2_-targeting strategies. ELISpot analysis of a wider range of cell lines revealed significant increases of IFN-*γ*-producing cells in cocultures with both G_D2_^int^ (TC-71) and G_D2_^low^ (VH-64, A4573, Cado-ES-1, WE-68) Ewing sarcoma cell lines (Figure 3B).

### G_D2_-specific, CAR-reengineered T cells exert potent cytolytic responses against Ewing sarcoma cells

**Figure 4:**
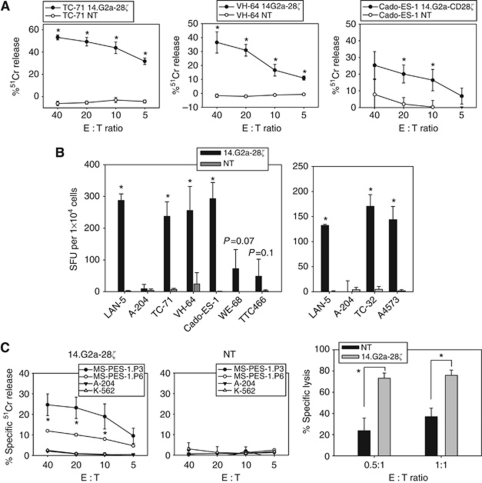
CAR-transduced, G_D2_-specific T cells exert cytolytic responses against allogeneic and autologous Ewing sarcoma targets. (**A**) Lysis of G_D2_^int^ (TC-71) and G_D2_^low^ (VH-64, Cado-ES-1) Ewing sarcoma cells by non-transduced (NT) and 14.G2a-28ζ-transduced T cells in a ^51^Cr release assay. Representative experiment of three. (**B**) Granzyme B secretion by 14.G2a-28ζ and non-transduced T cells in response to stimulation with various G_D2_-expressing target cell lines (see Figure 1 for G_D2_ expression levels). LAN-1 and A-204 cells served as positive and negative controls, respectively. Representative experiment of two. (**C**) Lysis of cultured tumour cells from a patient with relapsed Ewing sarcoma by autologous 14.G2a-28ζ and non-transduced T cells in a 4-h ^51^Cr release assay (left panels), and in a 16-h co-incubation assay by quantification of CD3-negative, CD45-negative, CD99+ tumour cells (right panel). Autologous cells after either three (MS-PES-1.P3) or six (MS-PES-1.P6) passages were used as targets in the ^51^Cr-release assay, and MS-PES-1.P6 cells were used for the 16-h co-incubation assay. Shown is one representative experiment of two. Asterisks indicate significant (*P*<0.05) differences to control.

In a 4-h ^51^Cr release assay, both VH-64 and TC-71 cells were efficiently lysed by 14.G2a-28*ζ*-transduced T cells (Figure 4A), and the lower G**_D2_**-expressing cell line Cado-ES-1 was also susceptible to lysis, albeit at lower efficiency. Significant granzyme B secretion by 14.G2a-28*ζ* gene-modified *vs* non-transduced T cells was found in response to both G_D2_^int^ (TC-71, TC-32) and G_D2_^low^ (VH-64, A4573, Cado-ES-1) cell lines, except for the lowest G_D2_-expressing Ewing sarcoma cell lines WE-68, TTC-466 where responses did not reach statistical significance. With all responsive cell lines, the quantities of response were comparable to those induced by LAN-5, regardless of G_D2_ expression levels, suggesting a threshold of G_D2_ expression necessary to trigger cytolytic T-cell responses via CARs (Figure 4B). Background responses of non-transduced T cells or of gene-modifed T cells to the G_D2_-negative target A-204 were negligible, confirming the specificity of the interaction. We conclude that the level of G_D2_ expression in many Ewing sarcomas is sufficient to specifically activate T cells and trigger cytolysis by granzyme B secretion via G_D2_-specific CARs.

### G_D2_-redirected T cells from Ewing sarcoma patients efficiently lyse autologous tumour cells

Translation of these findings into novel, G_D2_-based treatment strategies relies on the sensitivity of primary Ewing sarcoma cells to G_D2_ targeting and on the capacity of T cells obtained from sarcoma patients to mediate antitumour responses. Therefore, we performed autologous co-incubation experiments of short-term-cultured Ewing sarcoma cells from a relapse biopsy with 14.G2a-28*ζ*-transduced T cells, generated from peripheral blood after recovery of blood counts from chemotherapy. 14.G2a-28*ζ* transduced T cells efficiently lysed autologous Ewing sarcoma cells (Figure 4C). Remarkably, lysis of early passages of tumour cells was significantly higher compared with cells maintained in culture for further three passages. This observation is in line with the higher G_D2_ expression in early passages of cultured lines (Figure 1C) and the strong G_D2_ expression by immunohistochemistry in the majority of Ewing sarcoma tissue sections (Figure 2). In 16-h cocultures with autologous gene-modified T cells at 1 : 1 and 0.5 : 1 effector-to-target cell ratios, primary Ewing sarcoma cells were efficiently eliminated (Figure 4C). Thus, despite intensive cytotoxic pretreatment, functional G_D2_-specific T cells were efficiently generated from an Ewing sarcoma patient and potently and specifically eliminated autologous tumour cells *in vitro*, confirming the clinical feasibility of G_D2_ targeting.

### G_D2_-redirected T cells efficiently lyse Ewing sarcoma cells grown as spheres under anchorage-independent conditions

**Figure 5:**
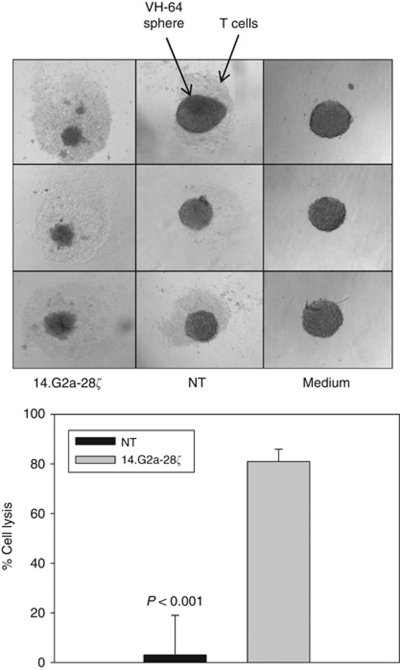
CAR-transduced, G_D2_-specific T cells efficiently lyse multicellular Ewing sarcoma spheres. Similar-sized VH-64 spheres of 200–250 *μ*m, reflecting about 1000 cells (Leuchte *et al*, manuscript in preparation) were co-incubated with 1000 14.G2a-28ζ T cells or non-transduced T cells per sphere, or in the presence of medium alone. Triplicates (shown in rows) of three pooled spheres each were used for analysis. The top panel shows photos of spheres following 16 h co-incubation. Spheres were then dissociated and stained with CD99-, CD3- and CD45-specific mAbs, followed by quantification of viable cells within the tumour cell gate by flow cytometry (bottom panel).

As monolayer cultures of Ewing sarcoma cells inadequately reflect the three-dimensional, anchorage-independent *in vivo* growth of tumour micrometastases (Lawlor *et al*, 2002), further experiments were performed in a sphere culture model. Ewing sarcoma sphere growth under restricted serum conditions has recently been shown to select for cells that possess higher tumourigenicity (Wahl *et al*, 2010). Moreover, we found that Ewing sarcoma spheres under these conditions are more resistant to chemotherapy than single-cell suspensions (Leuchte *et al*, unpublished data). Sixteen-hour cocultures of Ewing sarcoma spheres with G_D2_-retargeted T cells resulted in efficient lysis, whereas non-transduced T cells had no visible effect on sphere structure (Figure 5).

### *In vivo* antitumour activity of G_D2_-retargeted T cells against established tumour xenografts

**Figure 6:**
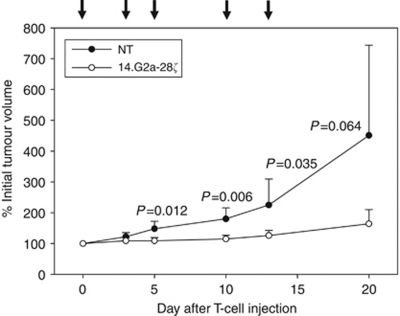
*In vivo* activity of 14.G2a-28ζ T cells against solid tumour xenografts. A cohort of 10 irradiated (3.5 Gy) NOD-scid mice were subcutaneously injected with 5 × 10^6^ VH-64 cells/mouse. Upon detection of palpable tumours, 5 doses of 1 × 10^7^ gene-modified, 14.G2a-28ζ T cells each were injected intratumourally over a period of 2 weeks (arrows). Control mice received analogous injections of non-transduced T cells. The analytic endpoint was relative tumour growth (in %), and was determined by dividing the tumour volume (mm^3^) at time of analysis by the initial tumour volume at onset of T-cell therapy (mm^3^) ( × 100).

To demonstrate the therapeutic efficacy of adoptively transferred 14.G2a-28*ζ* T cells, cohorts of 10 NOD/scid mice were subcutaneously injected with 5 × 10^6^ VH-64 cells per mouse. Upon detection of palpable tumours, five doses of 1x10^7^ gene-modified, G_D2_-specific T cells each were injected intratumourally over a period of 2 weeks. Control mice received analogous injections of non-transduced T cells. The analytic endpoint was relative tumour growth (in %). Mean tumour volumes at first injection of T cells were not significantly different between controls and 14.G2a-28*ζ*-treated mice (63.5±21.0 mm^3^ *vs* 82.6±38.0 mm^3^, *P*=0.40). Although tumours in mice receiving injections of non-transduced T cells continued to grow, 14.G2a-28*ζ* T cells efficiently and significantly reduced further tumour growth (Figure 6). Immunohistochemistry analysis of tumours removed from both 14.G2a-28*ζ* T cell-treated and control mice did not reveal any significant differences in G_D2_ expression (Supplementary Figure S4), arguing against either downregulation of G_D2_ or negative selection of G_D2_-negative or G_D2_^low^ cells under antigen-specific T-cell therapy. We further established an advanced vivo model that recapitulates the major aspects of the growth and progression pattern of disseminated Ewing sarcoma (Vormoor *et al*, 2001); however, unreliable engraftment of low cell numbers and highly heterogenous disease manifestations among mice intravenously injected with VH-64 cells impeded objective analysis of therapeutic effects.

Despite the limitations of our solid tumour model, the *in vivo* data demonstrate that the antitumour activity of G_D2_-retargeted T cells in Ewing sarcoma extends beyond single-cell suspensions and monolayers to both multicellular spheres and *in vivo* solid tumours.

## Discussion

The glycosphingolipid G_D2_ is an attractive target antigen for immunotherapy, as expression in normal tissues is highly restricted (Schulz *et al*, 1984). Moreover, G_D2_ is postulated to have a role in (tumour) cell adherence to the extracellular matrix (Cheresh *et al*, 1986) and may thus be involved in the local aggressive and metastatic phenotype of solid tumours. The prototype G_D2_+ malignancy is neuroblastoma, a paediatric solid tumour deriving from the sympathetic nervous system. Recent clinical phase II and III studies with G_D2_-specific mAbs (Yu *et al*, 2010), immunoconjugates (Shusterman *et al*, 2010) or retargeted T cells (Pule *et al*, 2008; Louis *et al*, 2011) have shown antitumour activity in high-risk neuroblastoma and are now starting to make an impact on the management of this disease.

Here, we provide evidence that Ewing sarcoma may be another candidate for G_D2_-targeting strategies. Although expression of G_D2_ in malignancies other than neuroblastoma, including melanoma (Tsuchida *et al*, 1989) as well as a proportion of high- and low-grade (non-Ewing) sarcomas (Chang *et al*, 1992; Modak *et al*, 2002), is not a novel finding, published data in Ewing sarcoma are limited. One study reports G_D2_ expression at low to moderate levels in 10 Ewing sarcoma cell lines (Lipinski *et al*, 1987), whereas another cell line was found negative (Schulz *et al*, 1984). In an analysis focussing on osteosarcoma, 2 of 4 Ewing sarcoma samples were G_D2_-positive (Heiner *et al*, 1987), and a primary Ewing sarcoma passaged in nude mice was reported to express G_D2_ (Cheung *et al*, 1987). Here, we substantially and systematically extend these findings and identify G_D2_ expression as a common feature of Ewing sarcomas including established cell lines, low-passage cell cultures, and primary biopsies. G_D2_ expression in Ewing sarcoma is compatible with the presumed cellular origin of the disease from either primitive neuroectodermal cells (Coles *et al*, 2008) or bone marrow-derived mesenchymal progenitor cells (Tirode *et al*, 2007), as both cell types express G_D2_ (Martinez *et al*, 2007).

G_D2_ expression in Ewing sarcomas was clearly not restricted to localised tumours or to primary diagnosis. These observations are in line with findings in neuroblastoma where G_D2_ is a reliable marker of disease throughout all stages and with consistent expression at relapse (Kramer *et al*, 1998). Future studies including large numbers of Ewing sarcoma patients should address the prognostic significance of G_D2_ expression status and include longitudinal analysis at both diagnosis and relapse. As G_D2_ surface expression is undetectable after paraffin-embedding of tumour tissue, cryopreserved material has to be systematically collected for these analyses.

From a clinical point of view, identification of G_D2_ as a reliable marker of Ewing sarcoma opens new avenues for therapeutic targeting. Here, we show that G_D2_-retargeted T cells efficiently interact with Ewing sarcoma cells and lyse tumour cells both *in vitro* and *in vivo*. Although Ewing sarcoma sphere structures were recently shown to provide resistance against cellular immune targeting by NK cells (Holmes *et al*, 2011), *in vitro* sensitivity to G_D2_-mediated T-cell lysis included multicellular sphere cultures grown under restricted serum conditions, modelling micrometastatic tumour growth. Moreover, evidence for an antitumour activity of G_D2_-specific T cells against larger and vascularised tumours was obtained *in vivo*. Thus, our findings strongly support translational application of G_D2_-targeted therapies in Ewing sarcoma.

Despite the low G_D2_ expression densities in many cell lines, the functional data presented here support effective interaction of a majority of Ewing sarcomas with G_D2_-redirected T cells. Interestingly, direct comparisons between early and later passages of two individual cell lines suggest some degree of downregulation of G_D2_ expression during prolonged *in vitro* culture. This observation, that has to be substantiated in a larger number of samples, may explain the discrepancy between the relatively high *in situ* expression of G_D2_ compared with established cell lines and further encourages therapeutic targeting of G_D2_ in Ewing sarcoma. A potential explanation is that differentiation-inducing agents present within the serum-containing culture medium may affect G_D2_ expression via transcriptional and/or epigenetic regulation of enzymes involved in ganglioside metabolism (Suzuki *et al*, 2011). The concern remains that subsets of G_D2_-negative tumour cells are present within Ewing sarcomas and may be selected for further tumour growth during G_D2_-targeted therapy. Besides clinical evaluation of G_D2_ targeting in Ewing sarcoma, elucidation of the significance of G_D2_ expression for the malignant phenotype of Ewing sarcoma will help to resolve the stability of this marker and its therapeutic usefulness.

As a carbohydrate antigen, G_D2_ is not presented to T cells by MHC class I. T cell targeting of G_D2_ has become possible by the CAR strategy that extends the target range of T cells to non-protein antigens (Rossig *et al*, 2001) and also bypasses the failure of many Ewing sarcomas to express HLA class I molecules (Berghuis *et al*, 2009). Over antibody targeting, T-cell-based approaches have several potential advantages. Firstly, T cells may establish an antigen-specific immune memory and provide long-lasting anti-tumour immune control (Dudley *et al*, 2002; Morgan *et al*, 2006; Bollard *et al*, 2007; Pule *et al*, 2008). Secondly, G_D2_-specific CARs lack the antibody Fc domain that is considered responsible for complement-dependent toxicities, including the neuropathic pain syndrome (Sorkin *et al*, 2010). Indeed, adoptive immunotherapy with 14.G2a*ζ*-transduced T cells was safe and well-tolerated in a first phase I study in neuroblastoma patients (Pule *et al*, 2008; Louis *et al*, 2011). Thirdly, CAR-reengineered T cells may overcome the limited avidity of mAbs against targets expressing low levels of antigen. The optimised design of current tumour-specific CARs and effective gene transfer techniques indeed enable responses to antigen^low^ targets (Ahmed *et al*, 2009; Yvon *et al*, 2009). As a consequence of the limited persistence, homing and antitumour function of CAR gene-engineered T cells in early clinical studies, current strategies focus on generating cells with robust capacity to expand, persist, and migrate to the tumour, and on delivering them into a supportive immune milieu. Some recent clinical studies have indeed demonstrated clinically significant antitumour activity of therapeutic T cells (Louis *et al*, 2011; Porter *et al*, 2011). One efficient means to improve expansion and persistence of transferred T cells appears to be the elimination of regulatory T cells by previous lymphodepleting chemotherapy (Dudley *et al*, 2002; Rapoport *et al*, 2011). In Ewing sarcoma, this can be achieved by applying T-cell therapy in the context of lymphopenia after high-dose chemotherapy with autologous stem cell rescue. Theoretically, off-target effects with G_D2_-positive normal bone marrow MSCs may interfere with haematopoietic stem cell engraftment in this context; however, no haematological toxicity was observed in any clinical trial in patients receiving G_D2_ mAbs or conjugates (Shusterman *et al*, 2010; Yu *et al*, 2010) or G_D2_-reengineered T cells (Pule *et al*, 2008). Increasing knowledge regarding local and systemic mechanisms of immune escape (Berghuis *et al*, 2011; Holmes *et al*, 2011) will further help to optimise integration of immunotherapies into the treatment of Ewing sarcoma.

In summary, this report extends the promise of G_D2_ targeting to the treatment of Ewing sarcoma. G_D2_-targeted immunotherapy may be a potent strategy to control or eliminate residual disease in patients with recurrent or refractory Ewing sarcoma.

## Figures and Tables

**Table 1:** Clinical characteristics and G_D2_ expression in tumour cell cultures and tissue sections

	**Age**	**Gender**	**Translocation**	**Primary tumour site**	**Primary metastases**	**Status**	**Tumour G_D2_**
*Neuroblastoma tissue sections*							
NBL-1							+++
NBL-2							++
	
*Tumour cell cultures*							
MS-PES-1	16	M	t(11;22)	Femur	None	DOD	positive
MS-PES-3	17	M	t(11;22)	Os sacrum	P	PD	positive
							
*Tumour tissue sections*							
1	15	F	t(11;22)	Ankle	P+O+BM	DOD	−
2	16	F	t(11;22)	Tibia	No	CR1 48 mo	++
3	7	F	t(11:22)	Pelvis	No	CR1	+/++
4	20	M	t(11;22)	Pelvis	No	CR1 56 mo	++/+++
5	15	M	t(11;22)	Pelvis, spine	P+B	DOD	+
6	28	M	t(11;22)	Pelvis	None	CR1 22 mo	++/+++
7	11	F	t(11;22)	Tibia	P	DOD	++
8	26	M	t(11;22)	Pelvis	No	Relapse	++/+++
9	23	M	t(11:22)	Humerus	BM, liver	DOD	−
10	19	M	t(11:22)	Humerus	P	CR1 18 mo	++/+++
11	17	M	t(11;22)	Clavicula	P	DOD	++/+++
12	16	F	t(11;22)	Kidney	No	CR1 62 mo	++/+++
13	13	M	t(11;22)	Pelvis	P	DOD	++/+++
14	12	F	t(21;22)	Fibula	No	CR1 53 mo	++

Abbreviations: BM=bone marrow metastases; CR=complete remission; DOD=dead of disease; F=female; M=male; mo=months; o=osseous metastases; P=pulmonary metastases; PD=progressive disease.

All patients had CD99+ small blue round cell tumours diagnosed as Ewing sarcomas by the reference pathology laboratory of the EURO Ewing 99 study center at Gerhard-Domagk-Institute of Pathology in Muenster, and all were treated according to this protocol. G_D2_ expression was determined by staining with FITC-labelled 14.G2a antibody and subsequent flow cytometry (cell cultures) or immunofluorescence analysis (tissue sections), respectively.
