# Supplementary material for: The ganglioside antigen GD2 is surface-expressed in Ewing sarcoma and allows for MHC-independent immune targeting
Source: Br J Cancer. 2012 Feb 28;106(6):1123–33. doi: 10.1038/bjc.2012.57 (PMC3304425; doi:10.1038/bjc.2012.57)
Supplement: Supplementary Figure S1 [file bjc201257x1.pdf]

Suppl. Figure S1

Cell line	D3S1358	vWA	FGA	Ameliogenin	TH01	TPOX	CSF1PO	D5S818	D13S317	D7S820	Cell bank profile available?*	
**CADO-ES-1**	16,18	14,18	21,22	x,x	6,9	8,11	11,12	11,12	10,12	11,13	Yes	100% Match
**SK-ES-1**	16,18	14,17	20,21	x,y	6,10	8,8	11,11	12,12	8,9	10,11	Yes	100% Match
**RD-ES-1**	15,15	17,17	21,25	x,y	7,8	9,11	11,11	11,11	11,12	10,10	Yes	100% Match
**TC-32**	15,16	15,18	23,24	X,X	6,10	9,11	11,13	12,13	10,12	8,11	No	Unique profile
**A4573**	16,17	18,19	24,24	X,X	7,8	8,10	11,13	11,12	8,10	11,11	No	Unique profile
**WE-68**	15,18	16,18	19,23	x,x	6,8	8,10	12,12	11,11	9,11	10,12	No	Unique profile
**5838**	16,16	15,18	20,22	x,y	10,10	8,8	11,11	10,10	13,13	11,11	No	Unique profile
**TTC-466**	17,18	15,17	24,25	x,x	7,7	8,8	10,10	10,10	10,12	8,10	No	Unique profile
**VH-64**	16,17	15,19	23,23	x,x	6,6	8,8	11,12	12,13	8,11	12,12	No	Unique profile
**MS-PES-1**	15,18	16,18	18,22	x,y	9,10	11,11	10,11	11,12	10,11	9,11	No	Unique profile
**MS-PES-3**	18,18	16,17	23,25	x,y	6,10	8,8	10,10	12,13	11,12	10,12	No	Unique profile
**TC-71**	15,17	17,18	24,26	x,y	10	8,9	10,11	10	11,12	10	Yes	100% Match
**Fibroblasts**	15,17	15,19	22,26	x,x	6,6	8,9	10,10	11,12	11,13	8,10	No	Unique profile
**LAN-5**	15,16	15,16	21,27	x,y	6,10	8,8	10,11	11,11	9,12	10,10	Yes	100% Match
**LAN-1**	16,17	16,19	19,24	x,y	8,10	8,11	12,12	12,12	11,12	10,11	Yes	100% Match
**JF**	15	16,19	23,25	x,x	7,8	8,11	11,12	12,13	13	11	No	Unique profile
**A204**	14,17	15,17	21	x,x	8,10	8,9	10,13	12	11,12	8,10	Yes	100% Match

*http://www.dsmz.de/fp/cgi-bin/str.html
