# Supplementary figures and images for: The ganglioside antigen GD2 is surface-expressed in Ewing sarcoma and allows for MHC-independent immune targeting
Source: Br J Cancer. 2012 Feb 28;106(6):1123–33. doi: 10.1038/bjc.2012.57 (PMC3304425; doi:10.1038/bjc.2012.57)

## Suppl. Figure S2

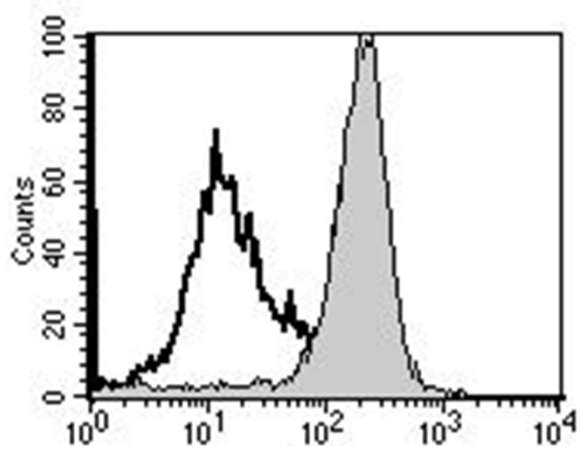

Anti-CD166 mAb 1172

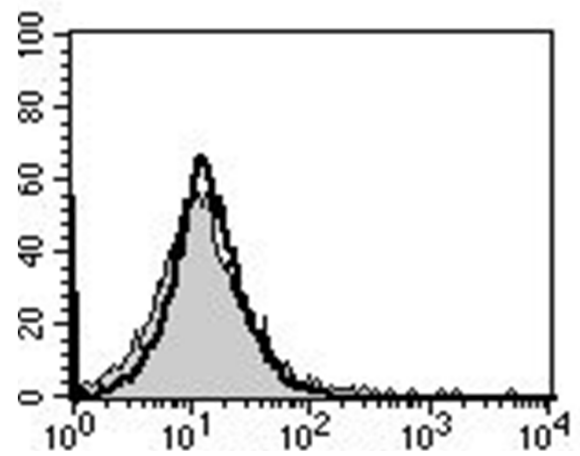

Anti- $G_{D2}$  mAb 14.G2a

Supplement: Supplementary Figure S2 [file bjc201257x2.pdf]

# Suppl. Figure S3

A

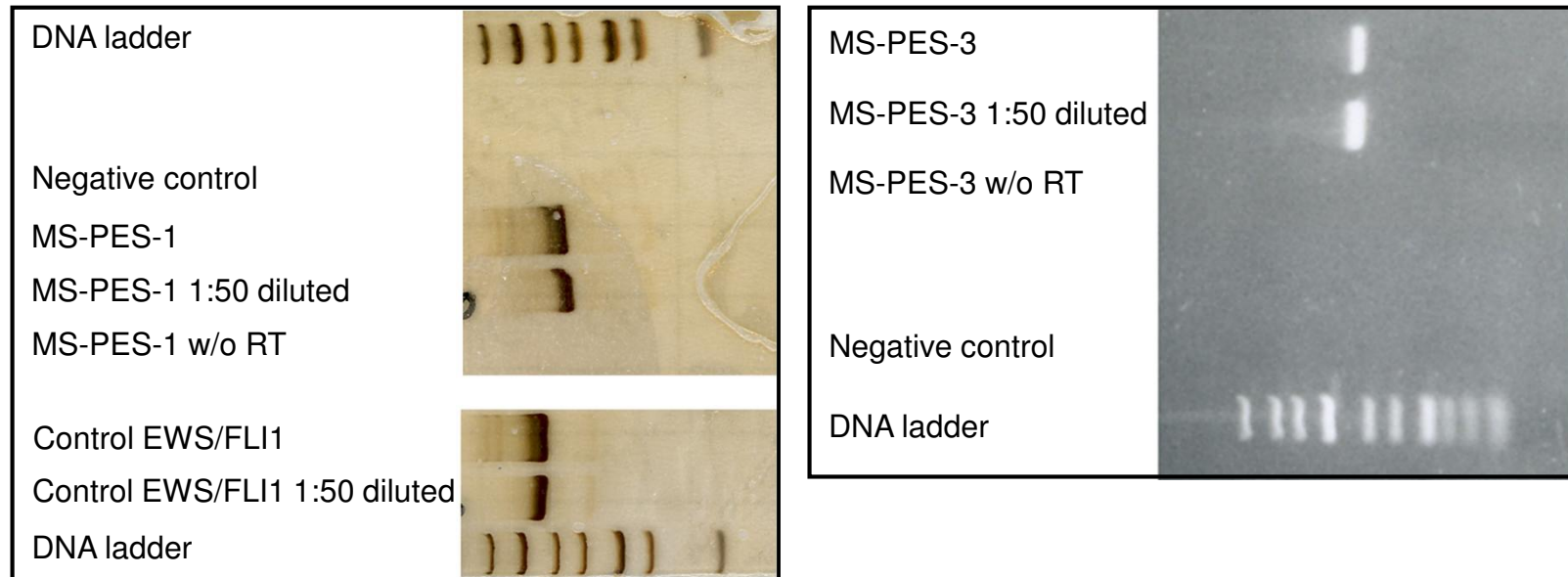

B

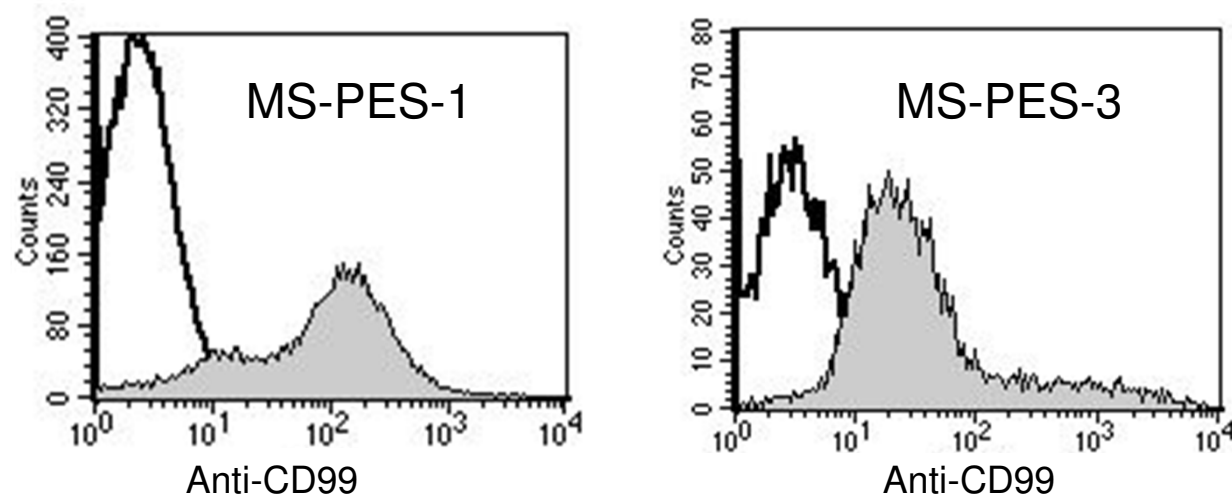

Supplement: Supplementary Figure S3 [file bjc201257x3.pdf]

Suppl. Figure S4

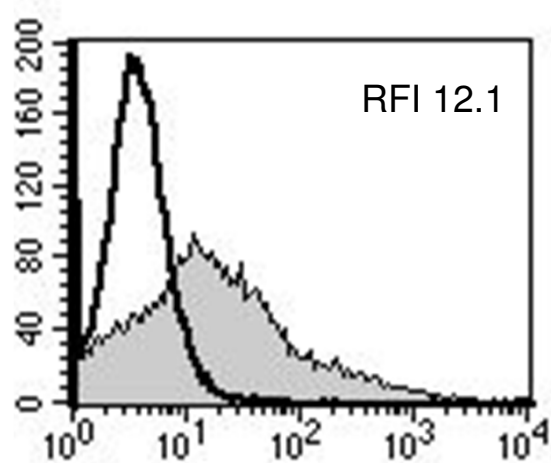

Cells from s.c. VH-64 tumor  
after 14.G2a-28 $\zeta$  CTL treatment

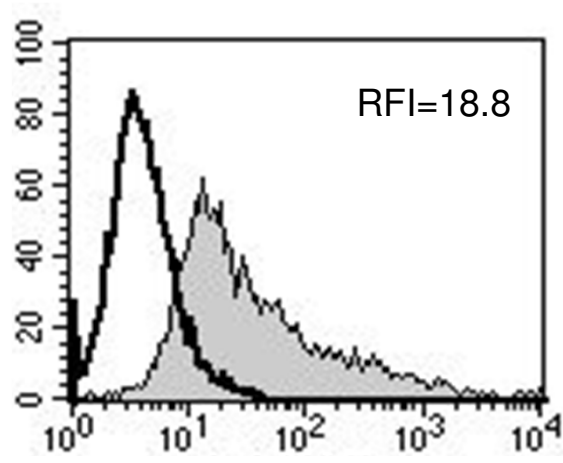

VH-64 original cell line

Supplement: Supplementary Figure S4 [file bjc201257x4.pdf]
