# Supplementary material for: The ganglioside antigen GD2 is surface-expressed in Ewing sarcoma and allows for MHC-independent immune targeting
Source: Br J Cancer. 2012 Feb 28;106(6):1123–33. doi: 10.1038/bjc.2012.57 (PMC3304425; doi:10.1038/bjc.2012.57)
Supplement: Supplementary Figure Legends [file bjc201257x5.doc]

**Legends to supplemental figures**

**Figure S1**. The identity of all cell lines was confirmed or established by short tandem repeat (STR) profiling

**Figure S2. 14.G2a does not cross-react with surface GD2.**

Human fibroblasts were stained with CD166-specific mAb 1172 and with 14.G2a.

**Figure S3. MS-PES-1 and MS-PES-3 are Ewing sarcoma cell cultures.**To confirm that the in vitro tumour cell cultures MS-PES-1 and MS-PES-3 generated from biopsy material at tumour relapse indeed represent Ewing sarcomas, we analyzed expression of the EWS/FLI-1 fusion transcript by RT-PCR, and surface expression of the Ewing-sarcoma associated antigen CD99 by flow cytometry. (A) Both tumour cell cultures express a characteristic EWS-FLI-1 fusion transcript, confirming the presence of the t(11;22) translocation. (B) Both cultures homogenously express surface CD99.

**Figure S4. GD2 expression is not downregulated upon GD2 targeting in vivo.**

Comparative analysis by flow cytometry of VH-64 cells resuspended from a tumor in a

14.G2a-28ζ treated mouse and the original cell line did not reveal any difference in GD2 expression.
